# The Validity of the American Diabetes Association’s Diabetes Risk Test in a Saudi Arabian Population

**DOI:** 10.7759/cureus.18018

**Published:** 2021-09-16

**Authors:** Faisal A Aldayel, Malak A Belal, Abdulrahman M Alsheikh

**Affiliations:** 1 Family and Community Medicine, Ministry of Health, Riyadh, SAU; 2 Medical Education, Prince Sultan Military Medical City, Riyadh, SAU; 3 Health Sciences, Johns Hopkins University, Baltimore, USA; 4 College of Medicine, Imam Mohammad Ibn Saud Islamic University, Riyadh, SAU

**Keywords:** glycated hemoglobin (hba1c), screening tool, prediabetes, diabetes, risk assessment, primary care, prediabetes screening

## Abstract

Introduction

The prevalence of type 2 diabetes (T2D) is growing worldwide. This study aimed to assess the sensitivity and specificity of the American Diabetes Association (ADA) and the United States Centers for Disease Control and Prevention's diabetes risk test in identifying Saudi Arabian patients at risk of developing T2D.

Methods

We conducted a one-month cross-sectional study that included patients older than 18 years who visited primary care facilities for any health concern in Riyadh City, Saudi Arabia. We used the Arabic language version of the ADA Prediabetes Risk Test questionnaire, a validated and reliable tool, to collect data. For this study, we analyzed the data using IBM SPSS Statistics for Windows, version 24.0 (IBM Corp., Armonk, New York). Moreover, we calculated sensitivity and specificity, positive predictive values (PPV), negative predictive value (NPV), the area under the curve (AUC), and Youden’s index.

Results

A total of 180 participants were included in the study (121 women and 59 men; mean age = 45 years). The ADA Prediabetes Risk Test sensitivity was 78.9, specificity was 82, PPV was 32, and NPV was 76. Youden’s index was 60.9 and the AUC was 0.6.

Conclusion

The ADA prediabetes risk assessment tool is highly sensitive and specific for determining the disease. It is a reliable and valid tool that has not yet been implemented to a great extent in Saudi Arabia. Therefore, future work should study the tool’s effectiveness in risk assessment in additional local Saudi Arabian communities.

## Introduction

The prevalence of type 2 diabetes (T2D) and prediabetes is increasing worldwide, and by the end of 2030, more than 470 million people could be affected by prediabetes globally, either because of insulin resistance or beta-cell dysfunction [1-4]. Prediabetes is a condition when blood glucose levels are elevated but remain below the threshold for a T2D diagnosis. The American Diabetes Association (ADA) defines prediabetes as fasting plasma glucose of 100-125 mg/dL (5.6-6.9 mmol/L), two-hour plasma glucose of 140-199 mg/dL (7.8-11.0 mmol/L), or glycated hemoglobin (HbA1c) of 5.7-6.4% (39-46 mmol/mol) [2,3].

T2D is an epidemic in Saudi Arabia, increasing in prevalence by 8% over the past 10 years and affecting 18.5% of the Saudi population [5]. According to the Saudi Arabian Ministry of Health, in 1992, 0.9 million individuals were diagnosed with T2D, and 2.5 million were diagnosed with T2D in 2010, revealing a 2.7-fold rise in prevalence in under two decades [6]. Approximately 40.3% of patients with T2D are unaware of their condition [6]. In Saudi Arabia, some attempts have been made to assess the population’s awareness and understanding of T2D risk factors and prevention strategies [7].

Several risk assessment tools have been developed worldwide, including the Australian Type 2 Diabetes Risk Assessment Tool [8], the Cambridge Risk Score [9], the Diabetes Algorithm, the Leicester Risk Assessment, and the Finnish Diabetes Risk Score (FINDRISC) diabetes risk calculator. A paucity of data exists on prediabetic risk assessment in Saudi Arabia, especially in patients visiting primary healthcare centers. Therefore, we conducted this study to assess the sensitivity and specificity of the prediabetes risk tool developed by the ADA and the United States Centers for Disease Control and Prevention to identify patients at risk of T2D visiting a primary healthcare facility in Riyadh City, Saudi Arabia.

## Materials and methods

We conducted a one-month cross-sectional study in July 2021 of patients aged 18 years or older visiting five primary healthcare facilities across Riyadh City, Saudi Arabia, using the ADA Prediabetes Risk Test questionnaire. The ADA quantifies diabetes status according to HbA1c levels, with normal as HbA1c <5.7%, prediabetes as 5.7% to 6.4%, and diabetes as 6.5% or higher.

The study included patients 18 years older. We excluded patients with a previous diagnosis of diabetes or pregnant and lactating women, or patients who do not need follow-up testing. The questionnaire used for the study is validated and reliable, and available in 15 languages, including Arabic, Urdu, English, French, Italian, Spanish, Japanese, and Korean [10]. The questionnaire records the patient's age, gender, family history, existing comorbid conditions, weight, height, and body mass index (BMI). The study was conducted following King Fahad Medical City Institutional Review Board's approval (21-268) and ethics committee's approval, and the ethical standards were kept intact during the entire period of study.

The estimated sample size was calculated using a power of 85% and a sampling error of 5% with sensitivity of the screening tool of 72% [11], and an estimated diabetes prevalence of 14.4% [12]. The minimum sample size needed is 176, which was increased by four patients to account for potential dropouts.

Study participants provided written informed consent for inclusion and self-completed the questionnaire during their health visit. We used IBM SPSS Statistics for Windows, version 24.0 (IBM Corp., Armonk, New York) to analyze the data. We used the Chi-square test and area under the curve (AUC) to assess the prevalence of prediabetes. We applied Youden's test to assess the questionnaire’s sensitivity and specificity.

## Results

Table 1 presents the patient demographics of the study population along with risk factors for T2D. Age does not seem to play a significant role in determining T2D risk factors. Women had a lower incidence of prediabetes than T2D, and men had more risk factors for the development of prediabetes than women.

**Table 1:** Characteristics of the sample according to prediabetes risks. Abbreviations: GD, gestational diabetes; HTN, hypertension; BMI, body mass index; ADA, American Diabetes Association.

Characteristics	Overall (n)	Euglycemic (n)	Prediabetes (n)	Diabetes (n)	P-value
Age					
Younger than 40 years	82	61	5	5	0.74
40-49 years	42	31	8	3	
50-59 years	28	20	8	0	
60 years and older	28	22	4	2	
Gender					
Women	121	91	22	8	0.502
Men	59	43	14	2	
Family history	85	59	19	7	0.215
GD					
No	15	12	3	0	0.614
Yes	165	122	33	10	
HTN					
No	34	25	9	0	0.082
Yes	146	109	27	10	
Physical activity					
No	33	22	6	1	0.703
Yes	147	108	30	9	
BMI (kg/m^2^)					
Low	47	38	5	4	0.32
Healthy	51	36	12	3	
Overweight	65	45	17	3	
Obese	17	15	2	0	
ADA score					
Normal	60	47	9	4	0.471
High	120	87	27	6	

Physically active patients and those with fewer ADA are at lower risk for prediabetes than those less active and those with more ADA scores. We found that 66.6% of people who do not engage in any physical activity do not develop T2D, but 73.4% of people who engage in a reasonable amount of physical activities do not develop T2D, indicating that physical activity help with T2D prevention. Hypertension (HTN) was not a dependent risk factor for T2D; the percentage of people getting T2D with HTN is the same as those getting T2D without HTN.

A family history of T2D is a risk factor for developing the disease. We found that 26% of people with a positive family history of T2D have prediabetes, while only 3% without a family history of T2D develop the disease (Table 1). People with greater ADA scores are more likely to have higher HbA1c levels and are thus more likely to develop T2D than those with low ADA scores.

Table 1 is also a comparative analysis of HbA1c and BMI scores. Patients with low or healthy BMI have less risk of developing prediabetes than those with high BMI.

Table 2 presents Youden’s index results. Youden’s index greater than 50 indicates reliability and validity to assess the risk factors for prediabetes. Youden’s index is significant enough to consider the ADA Prediabetes Risk Test reliable and valid. The AUC is 0.5; the test uses relevant risk factors that are predictive of prediabetes.

**Table 2:** Performance characteristics of the ADA Prediabetes Risk Test for detecting undiagnosed prediabetes in Riyadh, Saudi Arabia (n = 180). Abbreviations: ADA, American Diabetes Association; PPV, positive predictive value; NPV, negative predictive value; AUC, area under the curve.

ADA Diabetic Risk Test	High risk	Sensitivity	Specificity	PPV	NPV	Youden’s index	AUC
Cutoff at score >5	59	78.9	82	32	76	60.9	0.466
Cut off at score >4	64	81.7	70	34	78	51.7	0.466

Table 3 presents the number of valid positive (n = 134) and negative (n = 46) cases. Table 4 presents the AUC of 0.466 and the significance value of 0.491. The sensitivity of the prediabetic risk assessment tool was 78.9 when the cut-off was kept at five and 81.7 when the cut-off was kept at four. The specificity of the prediabetic risk assessment tool is 82 for the cut-off value of five and 70 for the cut-off value of an overall score of four. Moreover, both sensitivity and specificity are added together, and 100 is subtracted from the value to get Youden’s score. In this way, Youden’s score for the cut-off value of five was 60.9, and Youden’s score for the cut-off value of four was 51.7. The AUC for both cut-off values of five and four was 0.6. The positive predictive value (PPV) for the cut-off of five is 32, and the negative predictive value (NPV) for four is 76. Similarly, the PPV for the cut-off of four is 34, and the NPV for four is 78. The risk factor for the cut-off value of five has a high-risk value of 59, and the risk factor for the cut-off value of four has a high-risk value of 64.

**Table 3:** Case processing summary. ^a^True positives.

HbA1c	Valid (N, listwise)
Positive^a^	134
Negative	46
Missing	5

**Table 4:** Test result variables: ADA score. ^a^Accuracy of sample means. Abbreviations: ADA, American Diabetes Association.

Area under the curve	Standard error^a^	Asymptotic significance	Asymptotic 95% confidence interval	
			Lower bound	Upper bound
.466	.049	.491	.370	.561

## Discussion

This study sought to assess the ADA Prediabetes Risk Test in a Saudi Arabian population. Using the tool, we found several factors in our study population that affect the risk of developing prediabetes.

Risk factors in our population

According to the questionnaire results, men are more likely to develop the disease than women. This could be due to various factors, including the fact that visceral fat (more common to men than women) is a more reliable predictor for the risk factors for the development of prediabetes than BMI [13]. Men are more prone to smoking, alcohol consumption, lower education, and jobs that require less physical activity than women, which are also risk factors for prediabetes [13]. Another study has suggested that high testosterone levels are associated with T2D development [14]. Family history is a significant risk factor for developing T2D, and people with a family history are more exposed to developing the disease than those without it [15]. In addition, high ADA scores correlate with high HbA1c levels, so the association of ADA and HbA1c is a strong predictor for the development of prediabetes and T2D [15,16]. Therefore, our results align with those reported in the existing literature regarding T2D risk factors, which indicates the ADA tool is applicable in a Saudi Arabian population.

In low-income countries, HTN is common in adults and those with advanced age, and T2D is a chronic condition that creates the impression that people with HTN are likely to develop T2D; however, this is not accurate. The presence of one condition does not guarantee the presence of the other disease. People with HTN may avoid T2D if they adopt healthy living and proper lifestyle modifications at a young age [17].

Diet and lifestyle have a large impact on prediabetes development. Our results highlight that physical activity can reduce the risk of prediabetes and T2D and less physical activity is associated with a greater risk of developing the disease. Physical activity enhances metabolism and reduces glucose levels. All the risk factors in the assessment tool are interconnected. Healthcare professionals should focus on helping patients adjust modifiable risk factors to reduce T2D development.

According to our results, age has a lower impact on the HbA1c levels than other factors. Therefore, if patients adopt adequate lifestyle changes and dietary modifications, they may reduce their risk of T2D. However, we noted that participants in the age 60 years and older group had higher actual counts of T2D and prediabetes than expected. This may be because of an unhealthy lifestyle, negligence, or a lack of available preventive measures. Therefore, while age does not seem to play a significant role, advancement in age serves as a mild risk factor for the development of the disease.

Women with a history of gestational diabetes (GD) have higher risks of developing T2D than those without, especially if they do not adopt positive dietary and lifestyle modifications. In our study, the expected count for the onset of T2D in people with a history of GD is lower than the actual count, which means that GD is one of the most critical risk factors for prediabetes and T2D [18].

Risk assessment tool evaluation

In Saudi Arabia, there is no prediabetes tool approved by the Ministry of Health, and our results using the ADA risk tool reveal the prevalence of prediabetes and T2D in the country. The AUC value of 0.466 is significant enough to warrant using the ADA risk assessment tool in local Saudi Arabian communities.

The tool’s sensitivity is higher than the other tests being proposed in other studies [19], and this higher sensitivity is probably why this ADA test has been translated to many other languages to maximize its use. The high specificity and the sensitivity of the test are some of the primary reasons behind its accuracy. Any score above 50 for Youden’s index is a good score [19], which signifies the importance of the test and its reliability. The AUC of 0.6 signifies the reliability and authenticity of the test. Any value above 0.5 for the area under the curve is predictive of the test’s reliability and applicability [20].

PPVs in a screening test are the likelihood that a positive result correlates to an actual diagnosis, and NPVs are the likelihood that a negative test result correlates to a negative diagnosis of prediabetes. Our results suggest that higher NPV and lower PPV make the test reliable and authentic for broader use than our study population. The indication of risk value by the test reveals how beneficial it can be for the assessment of prediabetic risk factors.

Limitations

Our study was limited in its ability to account for other risk factors of the disease. Also, the study may have contaminated results because of the prevalence of type 1 diabetes. This lack of differentiation between the types may contaminate the results. Another important limitation is the large age range categories selected for the study. Young people might have different risk factors and different responses to those factors compared to older adults for whom those risk factors may vary in impact. Lastly, since the study was conducted in five primary healthcare centers in Riyadh City, our results cannot be generalized to a wider population beyond patients with access to those healthcare centers. Study results from primary healthcare centers located elsewhere may differ based on the sociodemographic and living standards associated with those regions.

## Conclusions

The ADA prediabetic risk assessment test is reliable, validated, and authentic in its screening for the risk factors for prediabetes and T2D in a Saudi Arabian population. Adequate information and more awareness of T2D can help prevent a rise in the prevalence of the disease. Most of the risk factors such as BMI and physical activities are modifiable risk factors, and therefore, dietary and lifestyle modifications can play a crucial role in bringing better health outcomes for the community.
